# Mechanisms of Kwashiorkor-Associated Immune Suppression: Insights From Human, Mouse, and Pig Studies

**DOI:** 10.3389/fimmu.2022.826268

**Published:** 2022-05-02

**Authors:** Husheem Michael, Joshua O. Amimo, Gireesh Rajashekara, Linda J. Saif, Anastasia N. Vlasova

**Affiliations:** ^1^ Center for Food Animal Health, Department of Animal Sciences, Ohio Agricultural Research and Development Center, The Ohio State University, Wooster, OH, United States; ^2^ Department of Animal Production, Faculty of Veterinary Medicine, University of Nairobi, Nairobi, Kenya

**Keywords:** human rotavirus, immunity, microbiota, protein calorie-deficient diet, gnotobiotic model

## Abstract

Malnutrition refers to inadequate energy and/or nutrient intake. Malnutrition exhibits a bidirectional relationship with infections whereby malnutrition increases risk of infections that further aggravates malnutrition. Severe malnutrition (SM) is the main cause of secondary immune deficiency and mortality among children in developing countries. SM can manifest as marasmus (non-edematous), observed most often (68.6% of all malnutrition cases), kwashiorkor (edematous), detected in 23.8% of cases, and marasmic kwashiorkor, identified in ~7.6% of SM cases. Marasmus and kwashiorkor occur due to calorie-energy and protein-calorie deficiency (PCD), respectively. Kwashiorkor and marasmic kwashiorkor present with reduced protein levels, protein catabolism rates, and altered levels of micronutrients leading to uncontrolled oxidative stress, exhaustion of anaerobic commensals, and proliferation of pathobionts. Due to these alterations, kwashiorkor children present with profoundly impaired immune function, compromised intestinal barrier, and secondary micronutrient deficiencies. Kwashiorkor-induced alterations contribute to growth stunting and reduced efficacy of oral vaccines. SM is treated with antibiotics and ready-to-use therapeutic foods with variable efficacy. Kwashiorkor has been extensively investigated in gnotobiotic (Gn) mice and piglet models to understand its multiple immediate and long-term effects on children health. Due to numerous physiological and immunological similarities between pigs and humans, pig represents a highly relevant model to study kwashiorkor pathophysiology and immunology. Here we summarize the impact of kwashiorkor on children’s health, immunity, and gut functions and review the relevant findings from human and animal studies. We also discuss the reciprocal interactions between PCD and rotavirus—a highly prevalent enteric childhood pathogen due to which pathogenesis and immunity are affected by childhood SM.

## Introduction

Malnutrition is commonly observed in children in developing countries and is a major cause of multiple illnesses. Kwashiorkor in children is presented with generalized edema and develops as a result of protein-calorie deficiency, while marasmus results from calorie and energy deficiency (1). In clinical practice, separating these two forms of malnutrition conclusively is difficult, which is further emphasized by the existence of marasmic kwashiorkor—a condition that combines different features of both (2, 3). Kwashiorkor represents a more severe and difficult-to-intervene condition as it contributes to secondary immune deficiency, intestinal dysbiosis, breach of epithelial barrier, growth faltering, and inflammation that further aggravates the immune dysfunction (4–7). Due to the breached epithelial barrier, kwashiorkor in children can also contribute to superimposed micronutrient deficiencies like, iron, zinc, selenium, and vitamin A (1).

Human rotavirus is the leading cause of death in children under 5 years of age (8). Two live attenuated oral vaccines are currently available, which are reported to be more effective in developed countries while their performance is suboptimal in the low- and middle-income countries (LMIC) (9, 10). Clinical studies have shown reduced seroconversion rates to oral vaccines in low-income settings, which is partially due to malnutrition/kwashiorkor in children (11, 12). Several attempts have been made to establish kwashiorkor models using mice and pigs. Pig model is the most biologically relevant non-primate model for transplantation of human intestinal microbiota (13–16). This is because of numerous similarities between humans and pigs in terms of their anatomy, physiology, and immune responses (14, 15, 17). Gnotobiotic (Gn) pigs have been extensively used to investigate human rotavirus infection and vaccine efficacy (18–22). Moreover, using a protein-calorie-deficient (PCD) diet in pigs transplanted with human infant fecal microbiota, a kwashiorkor model was developed that mimics major features of kwashiorkor in children (13, 14, 23–25). This model recapitulated compromised innate and adaptive immune responses and reduced effectiveness of human rotavirus vaccines. In this review, we have summarized the impact of kwashiorkor on children’s health, the associated immune deficiencies and infections, the effectiveness of oral vaccines, and the mechanistic insights into these interactions generated in Gn animal models of kwashiorkor.

## Childhood Malnutrition and Infectious Diseases

Malnutrition is the major public health concern affecting millions of people around the globe, especially those in countries with a low socioeconomic status. Impaired cell-mediated immunity, for example thymic atrophy, impaired tuberculin skin test, and reduced T-cell mitogenesis *in vitro* are the phenotypic characteristics of malnutrition (1). Malnutrition is common among children below 5 years of age. In 2010, malnutrition was estimated to affect close to 1 billion children globally, which is more than one-third of the worldwide childhood illnesses (26). According to the World Health Organization (WHO), malnutrition is a discrepancy between dietary intake of nutrients/energy in relation to the amounts required to maintain homeostasis and carry out specific functions including the linear growth in the case of children (1). A low socioeconomic status/poverty, inadequate dietary choices, physical and mental health illnesses, and proper nutrition unavailability are the commonly known causes of malnutrition. According to the WHO, stunting, wasting, being underweight, and micronutrient deficiencies are the main types of malnutrition, and each type originates from a distinct reason (1). Wasting and stunting exhibit a shared risk factor and often are evident in the same individual (27). Similarly, PCD diet resulted in weight loss, stunting, and growth failure in piglets and murine models (13, 28, 29).

**Figure 1:**
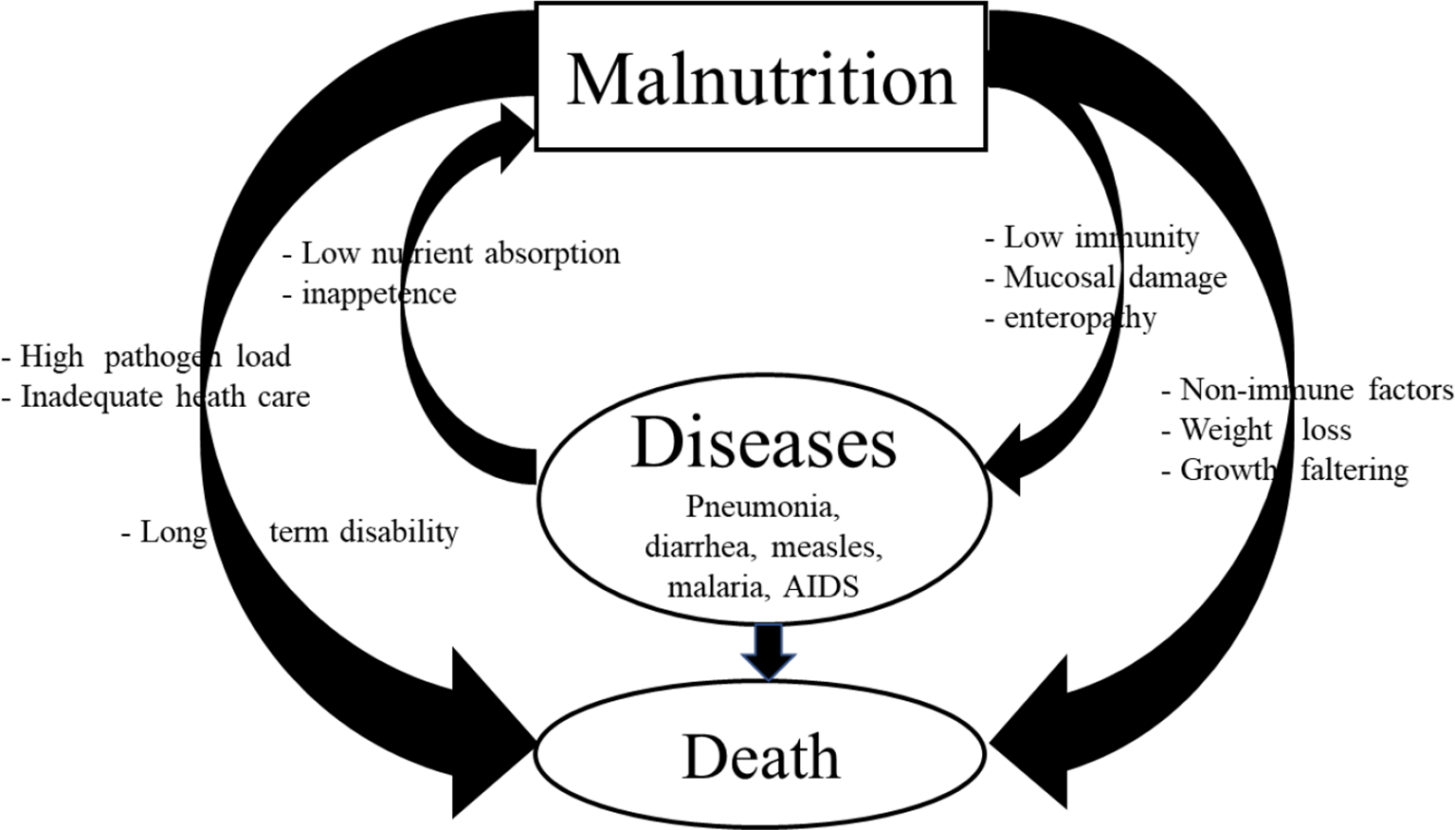
Vicious cycle of malnutrition and infection and its impacts on morbidity, mortality, and long-term disability.

Malnutrition represents a vicious cycle (30) and/or exhibits a bidirectional relationship with infectious diseases, whereby malnourished children are more susceptible to infections, and chronic or repeated episodes or persistent infections impair the digestive system to absorb nutrients leading to malnutrition that results in significant morbidity and mortality (**Figure 1**). An analysis of the findings from ten prospective studies performed in South America, Africa, and Asia revealed an association between the level of malnutrition and mortality (26, 31–33). These studies showed that even in the moderately malnourished children, wasting, stunting, and underweight resulted in significantly increased mortality from pneumonia and diarrhea. Moreover, there was an increased mortality risk among those with wasting more than among those with stunting (26). There is increased probability of mortality in children <5 years old with overwhelming anthropometric defects. Infectious diseases like pneumonia, influenza virus, rotavirus, malaria, human immunodeficiency virus, and measles account for more than 50% of all deaths in children worldwide (1). In addition, increased susceptibility to helminth, protozoan (*Cryptosporidium*, *Giardia*, *Entamoeba histolytica*), and tuberculosis (TB) infections is associated with malnutrition in children (1). Our studies using the Gn pig model demonstrated increased severity of rotavirus disease associated with intestinal dysbiosis and impaired immune function (13, 14, 23). Generally, undernutrition stems from a low socioeconomic status as a disadvantage that results in increased exposure to infections and increased mortality in malnourished children (34). Many of the deaths occurring among undernourished children are associated with chronic or repetitive infections.

## Kwashiorkor and Marasmus

Protein-energy malnutrition is a nutritional deficiency due to insufficient intake of protein and energy. It may have a broad range of clinical manifestations and frequently is accompanied by several micronutrient (like zinc, selenium, or iron) deficiencies. It can occur as acute, chronic, or acute–superimposed–chronic forms. The z-score [a precise measurement of deviation anthropometric measures (weigh/height for age) from the mean] was introduced to evaluate the severity of malnutrition. An acute form is characterized by low weight relative to height, while a chronic form (also known as stunting) is characterized by poor linear growth (length or height) against age (1). The WHO reference growth guidelines for sex and age are available for the grading of malnutrition into severe, moderate, or milder forms (www.who.int/childgrowth/standards/chart_catalogue/en/index.html). Practically, severe and mild acute malnutrition has a z-score of less and greater than -1, respectively. For chronic malnutrition, moderate malnutrition is a z-score between -2 and -3, while severe malnutrition (SM) would be a z-score greater than -3.

**Figure 2:**
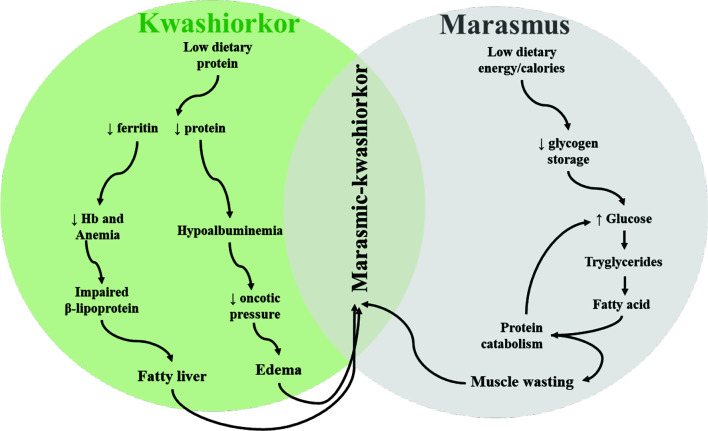
Schematic depiction of pathobiological features and interactions observed in different types of malnutrition (kwashiorkor and marasmus) in children.

***Severe acute malnutrition*** is normally classified as one of the two main conditions: kwashiorkor and marasmus. Marasmus is inadequate energy-calorie intake in all forms and is characterized by a weight-for-height value greater than 3 standard deviations and below the mean for sex and age. Children with marasmus appear to have lost subcutaneous adipose tissue and muscle mass through decreased glycogen storage (**Figure 2**). Marasmus children have a thin face with wrinkled skin, sunken cheeks, larger eyes, swollen abdomen, and sagging skin on the legs and buttocks. They appear older than their agemates and are irritable and have more appetite.

On the other hand, kwashiorkor is a protein deficiency which is independent of anthropometric values. Bilateral pitting edema of the face and lower extremities is one of the main clinical features of kwashiorkor. Other signs include total body edema (in severe cases), liver steatosis, body sores, depigmentation and sloughing of the skin (pale), thinning of hair, and inflammation, and children are apathetic and lose appetite (1, 35). Reduced protein intake leads to hypoalbuminemia that decreases oncotic pressure leading to edema (**Figure 2**). Similarly, hepatomegaly arises as a result of impaired synthesis of B lipoprotein due to reduced intake of proteins (**Figure 2**). If kwashiorkor is left untreated, it can lead to coma, shock, or death. Some studies have shown that not all severely malnourished children develop kwashiorkor; however, the reason behind this remains unknown (36). It is hypothesized that host genetics, microbiological factors, and other factors contribute to its development. The current advances in metabolomic, genomic, and immunological technologies may be helpful to unravel the mechanism behind this phenomenon (37, 38). Moreover, some children may develop marasmus-kwashiorkor-combined characteristics, with superimposed edema and severe wasting.

***Moderate malnutrition*** is characterized by a z-score between −3 and −2 standard deviation.

***Mild malnutrition*,** also called “at-risk” malnutrition, is defined as if any of the above-described indexes fall below 1 standard deviation below the median value for the reference population (35).

The mid-upper-arm circumference (MUAC) is a gauge of slim body mass that is associated with weight for height and a predictor of mortality risk (39). MUAC of <115 mm identifies severe acute malnutrition while ≥115 and less than 125 mm identify moderate acute malnutrition. Similarly, severe stunting is identified as a height for age more than 3 standard deviations less than the normal value for age or a height for age of <−3 standard deviation and correlated with height for age and MUAC (39). Precise nutrient evaluation is hardly ever done in the categorization of childhood malnutrition. However, children with evident anthropometric malnutrition exhibit or are prone to multiple nutrient deficiencies. Better characterization of the comorbidities associated with multiple nutrient deficiencies is needed (2).

Children with kwashiorkor are treated according to WHO regulations (40). Children with clinical signs and symptoms of kwashiorkor including evident infections, altered mental status, hypothermia, hypoglycemia, anorexia, and severe anemia are hospitalized, and nutritional rehabilitation is initiated and carried out in three phases. During the early phase, children with kwashiorkor are given a liquid therapeutic “F75”-formulated diet to fulfill daily requirements (100 kcal/kg/day) and other micronutrient supplements while preventing excesses of sodium and proteins. When the child is stabilized and expresses an appetite/able to eat, either “F100”-concentrated milk diet or ready-to-use-therapeutic food (RUTF) are given for a minimum caloric intake of ∼175 kcal/kg/day during the maintenance phase (40). RUTF is a diet enriched with high levels of lipid, protein, multivitamins, and micronutrients (41). Children with kwashiorkor with no clinical signs and symptoms can be directly treated with RUTF from the beginning if they express an appetite and able to consume the daily recommended calories (42–46). Recent data based on an evaluation of >20,000 kwashiorkor children observed that the therapeutic initiation of RUTF resulted in approximately 80% growth recovery among these children (47).

However, the malnutrition-induced phenotypic deficiencies are only partially restored by RUTF in a small percentage of receivers suggestive of very personalized responses that likely involve the gut microbiome. Recently, complementary microbiome-directed foods have been evaluated in preclinical studies in mouse and piglet models. These foods allowed to reestablish microbiome composition and function and increased biomarkers of normal growth, neurological development, bone remodeling, and immune function similar to healthy children (48).

Probiotics use has been associated with favorable outcomes in several clinical studies. The most common microorganisms used as probiotics are lactic acid bacteria, specifically of the *Lactobacillus*, *Enterococcus*, *Streptococcus*, *Bifidobacterium*, and *Pediococcus* genera, Gram-negative *Escherichia coli* Nissle 1917, and an yeast *Saccharomyces boulardii* (49, 50). The beneficial effects of probiotics have been extensively used to improve the host health and immune response, treat different infections including HRV, ameliorate irritable bowel symptoms, inhibit *Helicobacter pylori* replication, prevent cancer, reduce gastrointestinal inflammation, and prevent/treat allergies (50). More recently, “De Simone Formulation,” a combination of eight bacterial strains (four strains of *Lactobacillus*, three strains of *Bifidobacterium*, and one strain of *Streptococcus*), has been shown to improve intestinal epithelium function and ameliorate atherosclerosis, irritable bowel syndrome, ulcerative colitis, antibiotic-associated diarrhea, and radiation-induced enteritis (51). This further confirms that probiotics can be used for treatment of numerous infectious and inflammatory conditions and can benefit in malnourishment treatment.

Moreover, the Global Water, Sanitation, and Hygiene (WASH) Program (https://www.cdc.gov/healthywater/global/index.html) should be further promoted in Sub-Saharan Africa and Asia where malnutrition, poor sanitation, and water-borne illnesses are prevalent. The WASH program works on control measures for improving health and long-term disease prevention, reducing poverty, improving socioeconomic development, and responding to global emergencies including epidemics/endemics of life-threatening illnesses. These developments aim at reducing the lethal impact of WASH target diseases like diarrhea, cholera, typhoid fever, and hepatitis.

## Kwashiorkor-Associated Immune Suppression

In the malnutrition–infection interaction complex (**Figure 1**), malnutrition impairs immune function which further increases the incidence, severity, and duration of infectious diseases. Several models of this interaction/complex have been proposed over the decades, with current analysis that in addition to evident infections, enteropathy, subclinical infections, alterations in the gut microbiome function, and systemic inflammation are implicated in the pathogenesis of malnutrition (4–6). A detailed discussion of the impact of kwashiorkor on the immune impairment is presented as follows.

### Impaired Innate Immune Function

Dendritic cells (DCs) produce cytokines and present antigens to T cells, thus bridging innate and adaptive immunity. Hughes et al. demonstrated that Zambian children with kwashiorkor had normal numbers of white blood cells (WBCs), however, although the numbers of monocyte-derived DCs were reduced in their peripheral blood. The kwashiorkor-induced impairments were rescued following intervention using a protein-sufficient diet (52). Thus, they showed that antigen presentation to the cells of the adaptive immune system may have been reduced in kwashiorkor hosts due to decreased numbers of monocyte-derived DCs. Moreover, Nassar and colleagues reported that Fas (CD95/apoptosis antigen 1), a gene that signals to initiate apoptosis, is highly expressed in neutrophils, monocytes, and lymphocytes in kwashiorkor children indicative of impaired regulation of immunity and lymphoid homeostasis (53). However, this study showed that the expression of this marker was reduced following feeding a protein-sufficient diet, suggesting that the life cycle of WBCs is limited in kwashiorkor conditions.

The Kwashiorkor mouse model has shown anemia in addition to reduced numbers of lymphocytes, neutrophils, and monocytes (28, 29). In the kwashiorkor mouse model, peritoneal macrophages showed impaired phagocytosis (54, 55), reduced production of reactive oxygen species, and activation of nuclear factor-κB and nitric oxide (55) suggestive of reduced macrophage functionality. Additionally, Morris and colleagues showed that mice with acute kwashiorkor had decreased numbers of peritoneal macrophages, lymphocytes, granulocytes, serum albumin, impaired phagocytic activity, Th cells, and memory B cells, and all these effects coincided with significant weight loss. These impairments were restored by feeding these mice with protein-sufficient diets (56). In a neonatal kwashiorkor piglet model of rotavirus infection, we also observed reduced numbers of plasmacytoid DCs, CD103^+^ mononuclear cells, natural killer (NK) cells, and annexin V-positive apoptotic mononuclear cells and reduced gene expression by intestinal epithelial cells. These changes were associated with exacerbated human rotavirus infection and intestinal pathology evidenced by increased diarrhea and viral shedding, shortened villi, and increased lipopolysaccharide (LPS) levels indicative of impaired intestinal barrier function (13). Clinical studies have demonstrated decreased levels of acute-phase proteins (plasma C-reactive protein, haptoglobin, α_1_ acid glycoprotein, α_1_ antitrypsin) and pro-inflammatory cytokines (IL-6, IL-1, TNF-α) in severely malnourished children suggestive of inadequate immune function (57–60). Further, some studies demonstrated that malnourished children are capable of mounting an acute-phase response to infection that may sometimes occur in the absence of infection (6). Moreover, studies have shown low levels and dysfunctional responses of the C3 complement system which led to impaired phagocytic function of monocytes and lymphocyte function (reduced IL-1/IL-2 production) and decreased levels of several biochemical parameters, including total protein, albumin, prealbumin, and transferrin, in children with kwashiorkor (61–63). Overall, these changes are representative of immune suppression, immune dysregulation, and pro-inflammatory environment.

The neutrophil function in malnourished children is still not well defined. However, few studies have reported impaired neutrophil chemotaxis, microbicidal function, lysosomal enzyme synthesis, and decreased glycolytic activity in children with kwashiorkor indicating impaired cytolytic activity and weakened neutrophil functionality (64–66).

The numbers of NK cells in circulation were preserved in Ghanian infants (8–36 months old) suffering from moderate to severe malnutrition, while NK cell activity was reduced and subsequently rescued by nutritional intervention (67). Ritz and colleagues reported decreased NK cell numbers and cytotoxic function in the lungs and spleen of kwashiorkor mice after infection with influenza virus (68). In addition, our group had demonstrated that PCD decreased the function and frequencies of NK cell numbers, plasmacytoid DCs, CD103^+^ mononuclear cells, apoptotic mononuclear cells, and gene expression profiles of intestinal epithelial cells and increased serum LPS levels. These findings collectively establish suppression of the anti-infectious innate immune response and impairment of the gut barrier function in malnourished Gn pigs transplanted with human fecal microbiota, similar to observations in mice and children discussed above (13).

### Impaired Adaptive Immunity

The effect of malnutrition on adaptive immunity has substantial implications for the control of pathogens, response to vaccination, effectiveness of vaccines, and longevity of post-vaccine immunity. Deficiencies in adaptive immunity in undernourished children have been demonstrated in numerous studies. For example, peripheral CD8^+^ and CD4^+^ T lymphocyte numbers were preserved in malnourished children; however, in malnourished children there was no compensatory increase in B-cell frequencies following bacterial infections observed in well-nourished children (69). Interestingly, other studies demonstrated that malnutrition was associated with decreased numbers of effector (70) and memory (71) T cells. Consistent with these decreases, thymocyte depletion, thymic atrophy, and extracellular matrix alteration were observed in autopsies of malnourished children (72). Studies have shown that T lymphocytes are short-lived and dysfunctional in malnourished children. For instance, children with kwashiorkor and/or respiratory/gastrointestinal infections had increased apoptotic T cells, increased Fas (CD95) expression, and reduced levels of IL-7/IL-7 Rα and expressed inhibitory receptor-programmed death (PD-1) expression on T cells (73). PD-1 is known to inactivate T cells that fail to respond to infections and lead to reduced T-cell proliferation and IFN-γ secretion (74). Moreover, Fas and PD-1 pathways are implicated in the removal of infected T lymphocytes (73). Kwashiorkor Balb/c mice had splenic atrophy and variable splenic T-cell numbers (75, 76), while kwashiorkor rats had decreased frequencies of immature CD4^+^CD8^+^ T lymphocytes as a result of increased apoptosis of thymocytes and reduced lymphocyte proliferation (77). The above observations suggest that PCD led to altered thymus function. Moreover, T lymphocytes from malnourished children with bacterial infections had decreased levels of cytokines required for both Th_1_ differentiation (IL-12, IL-7, IL-21, IL-18) and function (IL-2, IFN-γ) (73, 78) but were characterized by overproduction of the Th_2_ cytokines (IL-10, IL-4) (79). This is suggestive of an impaired and imbalanced Th_1_ and Th_2_ immune response in malnourished children which results in failure to clear infections.

B-lymphocyte numbers and functions may be retained in kwashiorkor children; however, there could be alterations in specific antibody (Ab)-mediated immune responses. Najera and colleagues observed decreased numbers of B lymphocytes in kwashiorkor children with gastrointestinal (*Escherichia coli*, *Salmonella* spp., *Shigella* spp., and *Campylobacter* spp.) or respiratory (*Haemophilus influenzae*, *Streptococcus pneumoniae*, *Staphylococcus aureus*, and *Moraxella* (*Branhamella) catarrhalis*) infections compared with well-nourished children having similar infections (69). Consistent with observations in children, data from kwashiorkor C57BL/6J mice revealed that the Th_2_-type immunoglobulin (IgG1 and IgE) levels were elevated, while Th_1_-type immunoglobulin (IgG2a and IgG3) levels were maintained (80). However, IgG and IgM immunoglobulin levels were preserved in malnourished children with higher levels of IgA compared with well-nourished children (6). This likely was associated with skewing toward Th_2_ cytokines (IL-4, IL-10) and diversion from Th_1_ cytokines (IL-12, IL-2, IFN-γ) in malnutrition, indicating an impaired cell-mediated immunity incapable of eliminating pathogens. Additionally, el-Gholmy and colleagues observed a correlation between levels of IgA and the degree of dermatosis or edema in children with kwashiorkor; however, these findings have not been mechanistically explained (81). Recently, our lab has shown reduced titers of human rotavirus-specific IgG and IgA Ab in serum or intestinal contents and decreased numbers of Ab-secreting cells (ASCs) in blood and ileum as well as of CD8^+^ T cells (in spleen and ileum) and reduced inflammatory cytokine levels in a protein-deficient Gn piglet model (24, 25). These observations further confirm the PCD-induced impairment of adaptive immune responses following infections. Moreover, studies in humans and mice have reported that malnutrition impaired secretory IgA Ab production in response to various pathogens like typhoid, rotavirus, and cholera (82–86). Overall, these studies demonstrate that malnutrition impairs cell-mediated and humoral immunity toward intracellular and extracellular pathogens.

### Impaired Mucosal Barrier

The gastrointestinal epithelial barrier plays an indispensable role in the segregation of the interior of the mucosal layer from the luminal environment. Tight junctions expressed by intestinal epithelial cells are the key factors for building a barrier of epithelial cells and regulating the permeability of the barrier by tightly sealing the cell–cell junctions. Among tight-junction proteins, claudins play the most important component of the tight junction and are responsible for the barrier function and polarization of epithelial cells. Gastrointestinal diseases including infectious diarrhea, functional gastrointestinal disorders, reflux esophagitis, inflammatory bowel disease, and cancers may disrupt their functions leading to chronic inflammatory conditions or progressive diseases (87). The integrity and other functions of gastrointestinal mucosal and skin barriers are compromised in malnourished children and clinically evident in edematous malnutrition which is historically called “flaky paint” dermatosis of kwashiorkor (88). Originally, dermatosis has been described as effacement and atrophy of the skin layers and hyperkeratosis due to cutaneous inflammatory response, thus allowing the possible pathogen entry (89–91). Enteropathy has been observed in children with kwashiorkor characterized by villous atrophy, mucosal inflammatory infiltration, and impaired intestinal barrier function (7). Studies in Gambian infants (92, 93) have shown a correlation between growth faltering in infants and impaired small intestinal barrier function. Similarly, in our recent studies in Gn pigs, PCD resulted in decreased expression of the mRNA levels of the intestinal epithelial cell markers examined (MUC2, CgA, PCNA, SOX9, villin), which correlated with villous atrophy, and increased systemic LPS levels indicative of impaired intestinal epithelial cell function that contributes to stunting and increased rotavirus disease severity (13).

Recent investigations (94, 95) have emphasized the role of enteropathy, also known as environmental enteric dysfunction (EED)/environmental enteropathy (EE), in stunting malnutrition (96). Because of complications in procuring gut biopsies from infants, an effect of enteropathy on mucosal immune function remains unknown. It is further hypothesized that EED could be among the factors causing the inadequate efficacy of oral vaccines in impoverished countries due to impaired uptake of vaccine antigens (97).

The intestinal barrier impairment that ensues in the setting of enteropathy facilitates translocation of pathogens or their products into the systemic circulation. The latter leads to the following: (1) increased risk of Gram-negative bacteriemia in hospitalized kwashiorkor children (98); (2) higher levels of LPS which increase local and systemic inflammation and lead to impaired maturation of DCs, consequently being unable to efficiently maintain T-cell proliferation and function (52); and (3) increased stimulation of innate immune cells by infectious products that promote the pro-inflammatory environment in kwashiorkor children, which may contribute to suppression of the effector and regulatory T (Treg) cell functions. Moreover, studies have shown that PCD affected the morphology (reduced small intestinal villi height) and barrier/digestive function (increased serum LPS and reduced production of gastric enzymes) of the small intestine in piglet and rodent models (13, 99–101).

## Kwashiorkor-Associated Micronutrient Deficiencies

Kwashiorkor leads to several micronutrient deficiencies that are commonly overlooked; however, each micronutrient deficiency alone can cause important clinical disorders with profound alterations in the innate/adaptive immune function and host defense. Micronutrient deficiencies associated with kwashiorkor can possibly lead to synergistic or additive impairment of host defenses and may contribute to other illnesses (**Table 1**). In the following, we provide the short summary of the role of key micronutrients affected by kwashiorkor (iron, zinc, selenium, and vitamin A), in immune function.

**Table 1:** Micronutrient deficiencies associated with kwashiorkor and their effects on immune response in children.

Micronutrient deficiency	Effect on immune responses
Iron	• ↓ Phagocytosis • Impaired adaptive and innate immune response: ↓ IFN-γ; ↓ B/T cells; ↑ TNF-α; ↑ IL-6
Zinc	• ↓ Mucosal barrier function • ↓ Neutrophil function • ↓ Phagocytosis • Impaired Th_1_ and Th_2_ pathways • Thymic atrophy • ↓CD4/CD8^+^ T cells
Vitamin A	• Impaired innate immune response: ↑ necrotic MNCs; ↑ IFN-α; ↓ NK cells; ↓ CD103^+^ DCs; ↓ TLR3 • Impaired adaptive immune response: ↑ CD8^+^ T cells; ↑ IFN-γ; ↑ IL-12; ↓ IL-10; ↓ FoxP3 T regulatory cells
Selenium	• Impaired T cell-dependent Ab responses • ↓ Phagocytosis • Impaired T cell functions: ↓CD3+ cells; ↓IL-2; ↓NFAT

MNCs, mononuclear cells; NK, natural killer; TLR, toll-like receptor; Th, T helper; DCs, dendritic cells; NFAT, nuclear factor for activated T cells; ↓, reduced; ↑, increased.

***Iron*:** Iron deficiency is widespread in LMIC causing hypochromic anemia, developmental delays, fatigue, cognitive defects, and behavioral abnormalities (1). The role of iron in both innate and adaptive immunity is invaluable. Moreover, iron deficiency results in the impaired killing of bacteria by phagocytosis which leads to excessive pathogen replication (102). Iron deficiency results in impaired phagocytosis which leads to the reduction of myeloperoxidase-containing granulocyte impairing cytotoxic function of macrophages in anemic children (103). On the other hand, iron overload increases the risk of cellular toxicity and increases the risk of infections like TB or malaria (104, 105). Peripheral blood mononuclear cells from iron-deficient children showed increased IL-6 and TNF-α mRNA expression levels following iron supplementation (106), implying that iron deficiency impairs the homeostasis of these cytokines. Moreover, iron-deficient mice had reduced secretion of IFN-γ by mitogen-activated splenocytes, suggesting that T-cell effector function is impacted by inadequate iron levels (107). Consistent with the above, transferrin receptor 1 (TfR1)-deficient mice, characterized by decreased cellular iron uptake, showed weakened T- and B-cell responses compared with control mice (108). In addition, mice with conditional deletion of ferritin H had reduced T- and B-lymphocyte frequencies in their lymphoid tissues (109). Similarly, the TfR1-blocking antibodies reduced the proliferation of human T and B lymphocytes (110). Recently, it has been demonstrated that anemia due to deficiency of iron at the time of immunization resulted in reduced responses to vaccine against diphtheria, pertussis, and pneumococcus; however, the humoral vaccine response to these diseases increased with administering iron supplement together with the vaccine (111), further emphasizing the role of iron in immunity. Moreover, reduced hematocrit and hemoglobin levels were observed in a PCD piglet model consistent with clinical signs of iron-deficiency anemia (99). Altogether, it emphasizes the importance of adequate iron levels for adaptive immunity.

***Zinc*:** Low zinc levels are implicated in cellular growth retardation, while excessive zinc is toxic to the cellular environment (112). Zinc deficiency usually occurs in children with kwashiorkor especially in low-income countries, while PCD (vegetarian) diets are known to lead to zinc deficiency in murine models (113–116). Zinc deficiency may result in impaired mucosal immune function through altered epithelial homeostasis (1): imbalanced Th_1_ and Th_2_ immune responses (117), reduced phagocytosis by macrophages, and impaired NK-cell function and neutrophil dysfunction (118, 119). Moreover, deficiency of zinc may cause increased apoptosis of double-positive (CD4^+^CD8^+^) immature T cells because of thymocyte apoptosis (120) resulting in reduced synthesis of Th1 cytokines (IL-2, IFN-γ). Impaired humoral and cellular immune responses were observed in zinc-deficient mice after vaccination against hepatitis B (121). Additionally, zinc deficiency can alter the composition of the microbiota resulting in dysbiosis due to a direct competition between intestinal bacteria for zinc availability (122). These authors demonstrated that while no specific immune-mediated mechanisms were identified, *C. jejuni* was unable to replicate or colonize the gastrointestinal tract without the high-affinity zinc transporter. Additionally, under minimal zinc conditions, studies demonstrated an increased risk of gut colonization by *Haemophilus* spp., *Salmonella enterica* serovar *Typhimurium*, *C. jejuni*, and *E. Coli* (123–126). Medeiros and colleagues showed that biofilm formation, as well as epithelial cell binding and expression of virulence factors by enteroaggregative *E. coli* reduced by zinc supplementation of zinc-deprived mice, results in decreased fecal shedding and rescinded growth stunting (127). Moreover, zinc supplementation can beneficially alter intestinal bacterial composition *via* competitive exclusion between the invading pathogens and the host commensal bacteria (128, 129); treat diarrhea and pneumonia; and decrease the risk of infections (117). Moreover, parakeratosis (zinc-responsive dermatosis) was observed in zinc-deficient piglets exhibiting diarrhea, vomiting, anorexia, and high mortality (130), and the clinical symptoms were reversed following zinc supplementation (131).

***Selenium*:** Selenium serves as an antioxidant, and its deficiency increases oxidative stress that conversely impacts immune cells during activation, differentiation, and proliferation. Selenium deficiency is generally observed in children with kwashiorkor in geographical regions where soil is deficient in selenium (132). In addition, kwashiorkor children are more prone to develop selenium deficiency than marasmus children due to impaired gut barrier or other unknown mechanisms (133). Selenium is a vital regulator of major metabolic pathways, and it exhibits strong anti-inflammatory properties (134). T cell-dependent antibody responses were impaired when the selenocysteine tRNA gene was deleted in knockout mice resulting in fewer functional T cells that led to excessive reactive oxygen species (ROS) production and an impaired macrophage function manifested by higher ROS levels that altered the regulation of extracellular matrix (ECM)-related gene expression and reduced the migration of macrophages in a protein gel matrix (80, 135). These authors demonstrated that selenoprotein-deficient macrophage–ECM interactions were distorted by the absence of selenoprotein expression in such a way that ECM remodeling was decreased, and the surrounding ECM was fortified. The latter could have impaired the migration of macrophages through the ECM and basement membrane, thus indicating an impaired phagocytosis and failure to clear viral infection (80).

In addition, dietary selenium deficiency results in impaired calcium mobilization, neutrophil function, and reduced nuclear factor of activated T cells (NFAT) (136–138). Calcium mobilization is required for T-cell proliferation, and proliferation of both CD4^+^ and CD8^+^ T cells was decreased in selenoprotein K (Sel K) knockout (Sel K^-/-^) mice. Moreover, T cell migration, induced through chemokine (SDF-1 and RANTES) receptors and dependent on efficient calcium flux, was significantly decreased in Sel K^-/-^ T cells (136). Chemotaxis of neutrophils relies on effective calcium release from the endoplasmic reticulum upon chemokine receptor engagement. Studies have demonstrated that the frequency of neutrophils in bone marrow and the expression of the chemokines KC and CXCR2 were similar between wild-type and Sel K^-/-^mice (133). However, neutrophils’ ability to migrate in response to KC was significantly decreased in neutrophils from Sel K^-/-^ mice (136). Finally, another study demonstrated a dose-dependent increase in NFAT translocation (a critical signaling event downstream of calcium flux) following dietary selenium supplementation (139). Taken together, it indicates that dietary selenium is indispensable for efficient immune functions.

Janbakhsh and colleagues observed enhanced immune responses against hepatitis B vaccine in insulin-dependent diabetes mellitus patients supplemented with selenium (140). We have observed a compensatory increase in serum selenium and vitamin A&E levels suggestive of these micronutrient deficiency in PCD piglets not observed when micronutrient homeostasis is maintained (13). Moreover, a selenium deficiency-mediated increase in mortality rates was observed in piglets, while selenium supplementation reduced the mortality rates, once again emphasizing its importance for immunity and health (141).

***Vitamin A (VA)*:** VA is essential for the growth and development of children, and its deficiency results in profound defects of the innate and adaptive immunity. Vitamin A deficiency (VAD) is often found in children with kwashiorkor due to the inadequate availability of retinol-binding protein (RBP, major protein in vitamin A transfer) in the liver. Millions of children in impoverished countries are affected by VAD resulting in impaired mucosal immunity and oral vaccine ineffectiveness (142, 143). Our previous studies in the neonatal VAD Gn pig model have demonstrated impaired efficacy of human rotavirus vaccines evidenced by increased HRV shedding, diarrhea severity, and decreased immunoregulatory cytokine (including IL-10) production (144, 145). Moreover, we demonstrated that prenatally acquired VAD altered innate immune responses by increasing necrotic annexin V-negative mononuclear cell numbers, while reducing IFN-α levels (post-HRV challenge) and frequencies of CD103-expressing DCs and TLR3^+^ mononuclear cells that led to increased HRV shedding and diarrhea (144). CD103^+^ DCs convert CD4^+^ T cells into FoxP3^+^ Treg cells and induce gut-homing phenotypic changes of lymphocytes (146). Further, while IFN-α and increased TLR3 expression are associated with improved RV clearance, VAD modulates these innate immune aggravating HRV infections (144). Additionally, using the Gn pig model, we showed that VAD led to impaired adaptive immunity skewed toward a pro-inflammatory state characterized by increased CD8^+^ T cells, IFN-γ levels, and IL-12 (suggestive of Th_1_ microenvironment) and reduced levels of anti-inflammatory cytokine IL-10 as well as numbers of FoxP3^+^ Treg cells (147). Since IgA production and B-cell functionality are dependent on VA availability, VAD reduced HRV-specific IgA antibody-secreting-cell numbers and intestinal B-cell frequencies, thus increasing HRV shedding/diarrhea and systemic IFN-γ levels (147). Moreover, Iwata and colleagues demonstrated that VAD reduces the expression of the gut-homing molecules α4β7 and CCR9 on CD4^+^ T cells in a murine model, thus reducing protection against mucosal pathogens (148). Retinoic acid (a biologically active VA metabolite) stimulates CD103^+^ DCs to imprint α4β7 and CCR9 on B/T cells that signals homing to intestinal mucosa, stimulates IgA Ab secretion, and restores NK-cell function essential for HRV clearance and protection against HRV diarrhea (148–150). Spencer and colleagues have observed that VAD reduced type 3 innate lymphoid cells (ILC3) in a transgenic murine model which resulted in compromised immunity following acute enteric bacterial infection (151). ILC3 produces IL-22 whose main function is to maintain intestinal health through regulation of the intestinal barrier and commensal microbiota and act as an antigen-presenting cell to promote protective immune responses against extracellular microbial pathogens (152). It has been demonstrated that ILC3 development and functionality are dependent on RA stimulation of the ILC3 master transcription factor (RORγt) (153). VAD also impacted the number and function of NK cells in humans and rodents (154, 155). NK cells are innate immune cells that exhibit effective cytolytic activity against virus-infected cells and play a crucial role in the regulation of early immune response to viral infections by secreting proinflammatory cytokines (TNF-α, IFN-γ) that further modulate the function of other innate and adaptive immune cells (156). Additionally, NK cells engage in reciprocal interactions with dendritic cells, macrophages, T cells, and endothelial cells playing an immunoregulatory role (157). Thus, impaired function of NK cells compromises antiviral defense directly and indirectly *via* limiting or exacerbating other immune responses. In another study, VAD significantly altered bacterial diversity and meta-transcriptome response determined by the AcrAB-To1C efflux system and was found to increase the relative abundance of *Bacteroides vulgatus* spp (158).. *B. vulgatus* is a growth-discriminatory strain identified in a preclinical Gn mouse model of human gut microbiota development (159). The underrepresentation of this growth-discriminatory strain in the gut microbial communities of malnourished children gives a rationale for VA interventions targeting the abundance and functions of this bacterial species (160).

Taken together, this information regarding the effects of kwashiorkor-induced micronutrient deficiencies provides mechanistic understanding of the associated pathological immunophenotype. This knowledge is also critical for further improvement of the existing approaches of the micronutrient deficiency management.

## Kwashiorkor-Associated Microbiome Alterations

Intestinal microbiota regulate the host metabolism and thus play an important role in promoting and maintaining human health (161). Intestinal microbiota induce and regulate immune maturation, induce immune tolerance toward self and food antigens, and strengthen host defense and homeostasis in recuperation from intestinal infections (162). Biotic or abiotic stresses reduce the functions of the microbiome and the amounts of beneficial metabolites available for the host (161). A study has shown that the composition and activities of the intestinal microbiota can orchestrate several local and systemic functions (163).

There exists a tri-directional relationship among intestinal microbiota, host epithelial cells, and immune response; therefore, alterations of microbiota composition in the undernourished host can lead to dysregulation of intestinal mucosal immune function leading to enteropathy and increased susceptibility to infections (164–166).

Studies from Bangladeshi children revealed that the kwashiorkor-associated fecal microbiota was significantly less diverse (immature) compared with that of age-matched healthy children (167). However, this condition was reversible and the microbiome composition has been restored to the diverse (mature) phenotype when these kwashiorkor children were given RUTF and treated with antibiotics (167). Moreover, studies from Bangladesh and Malawi kwashiorkor children revealed an association between low microbiota diversity, increased relative abundance of *Acidaminococcus* spp., and growth faltering (168). It was suggested that *Acidaminococcus* spp. reduced the availability of glutamate, a vital amino acid required for the homeostasis of the intestinal epithelium. It is further hypothesized that overgrowth of other glutamate-fermenting bacteria could be the reason for the stunting. Besides, glutamate plays a key role in the induction and development of T-cell-mediated immunity in peripheral tissues and in the central nervous system (169). A study from India demonstrated that the kwashiorkor-associated dysbiosis/relative reduction of specific genera (*Roseburia*, *Butyrivibrio*, *Faecalibacterium*, *Phascolarctobacterium*, and *Eubacterium*) coincided with vitamin, essential fatty acid, and various mineral deficiencies in children (170). In Uganda, a cohort study observed reduced fecal microbiota diversity in marasmus children compared to children with kwashiorkor but failed to identify specific distinctions in abundance among individual genera in kwashiorkor vs. marasmus children (171). Some previous studies have reported that microbiota of malnourished children may contain increased numbers of pathogens or pathogenic virulence factors (170, 172); however, this was challenged by some subsequent studies (167, 173). An association between altered gut microbiota and retarded growth was demonstrated in Malawian twins discordant for kwashiorkor (173). To demonstrate the contribution of the dysbiotic gut microbiome to kwashiorkor development, the fecal microbiota from the well-nourished and malnourished twins were transplanted to Gn mice fed with nutrient-deficient Malawian diet. The combination of the malnourished twin microbiota and the nutrient-deficient diet resulted in the most marked weight loss in the mice, suggesting that dysbiotic intestinal microbiota contribute to kwashiorkor pathobiology (173). Stunting of mice that received microbiota from children with kwashiorkor could be rescued/prevented by the co-transfer of *Clostridium symbiosum* and *Ruminococcus gnavus* spp. present in the microbiota of well-nourished children (159). *Ruminococcus gnavus* is an early colonizer of the gut (174) that persists in healthy adults as it was detected in more than 90% of human fecal samples by metagenomic sequencing (175). *Ruminococcus gnavus* degrades/converts complex polysaccharides into a diverse nutrient (159). *Clostridium symbiosum* is a non-toxin-producing strictly anerobic bacterium present in the normal gastrointestinal and vaginal bacterial microbiota of humans and animals and that plays a role in glutamate metabolism. Both species act as lean mass gain discriminatory taxa (159, 176).

The above microbiota alterations lead to permeability of the intestinal epithelial barrier, enabling the translocation of intestinal pathobionts into local and systemic tissues induced by PCD diet which were enhanced following human rotavirus infection (14, 177–179). Recently, we have demonstrated that PCD diet reduced the ratio of *Firmicutes* to *Bacteroidetes* in neonatal Gn piglets (180). An increased *Firmicutes*-to-*Bacteroidetes* ratio is characteristic for obesity while a reduced ratio is associated with inflammatory conditions (181). Moreover, PCD correlated with higher levels of *Proteus* in the duodenum and jejunum, whereas the relative abundance of *Turicibacter* was reduced in spleen and mesenteric lymph nodes (180). The relative abundance of *Proteus* species was shown to correlate with pro-inflammatory (TNF-α, IL-1β) responses in the pathogenesis of gastrointestinal illnesses including ulcerative colitis and Crohn’s disease (11); while the relative abundance of *Turicibacter* species correlated with effective T cells immune function in rodents (12, 182).

Nutrients can modulate intestinal inflammation either directly through the ligand–receptor interactions or indirectly through the alteration of the microbiota and by-products of their metabolism. Some specific nutrient deficiencies can also cause dysbiosis resulting in impaired mucosal immune homeostasis (183). For instance, the transport of dietary tryptophan through the angiotensin I-converting enzyme in the small intestinal epithelium increased the generation of antimicrobial peptides, the intestinal epithelial barrier, and the composition of the intestinal microbiota and decreases the susceptibility to intestinal inflammation in Gn mice (184). Recently, our group has demonstrated that PCD diet reduced tryptophan and angiotensin-converting enzyme 2 (ACE2) levels which coincided with impaired activation of adaptive immune responses following human rotavirus infection in the Gn piglet model (23). Kuba et al. have demonstrated that ACE2 is a vital receptor recognized by severe acute respiratory syndrome coronavirus (SARS-CoV) and SARS-CoV-2 (185) and a negative regulatory factor for severity of lung edema and acute lung failure (185). Therefore, it is likely that kwashiorkor may worsen the health outcomes of COVID-19 infection. Furthermore, children with kwashiorkor exhibit a reduced breakdown of body proteins, which reduces the availability of essential amino acids leading to a reduced production of plasma proteins critical for the acute-phase response to infections, immune responses, and nutrient transportation (58).

As noted above, kwashiorkor in children causes intestinal dysbiosis and hence they are prone to exhibit bacterial infections; therefore, a short course of antibiotics use has been recommended for these children. The recommended guideline regarding the use of antibiotics for children with kwashiorkor was revised in 2014 (42). A study conducted in Malawi, where kwashiorkor children (6 to 59 months old) were treated for 7 days with cefdinir, amoxicillin, or placebo in combination with RUTF, showed that children that received RUTF and antibiotics had accelerated weight gain, decreased mortality rates, and increased recovery rates than those who received placebo (186). While there are no data on the *Firmicutes*-to-*Bacteroidetes* ratio in recovered kwashiorkor children treated with RUTF and antibiotics, previous studies suggest that the effects may be antibiotic-specific (187).

## Kwashiorkor Impacts on Infection and Vaccination: A Case of Rotavirus

As discussed above, the malnutrition-infection cycle is complex, and it seems obvious that vaccinations in early childhood may prevent infections, thus lessening the impact/severity of malnutrition (188).

Adequate dietary intake primarily defines the diversity and functions of the intestinal microbiota and epigenetically regulates the host metabolism by DNA methylation that consequently is advantageous in long-term health (189–192). Rotavirus is a leading cause of childhood diarrhea especially in developing countries. In 2000, it was estimated that over half a million children died before their 5th birthday due to rotavirus-associated gastroenteritis (8). In the Global Enteric Multicenter Study, it was noted that rotavirus-associated diarrheal illness was caused by malnutrition (193). Rotarix^®^ and Rotateq^®^ are two live oral rotavirus vaccines approved by the FDA, and their efficacy against severe rotavirus infection was shown to be 85%–98% in developed countries. However, their efficacy in low- and middle-income countries only reached 48%–64% (9, 10). As noted, children with kwashiorkor exhibit intestinal dysbiosis, altered metabolism, EED, epithelial barrier dysfunction, and innate/adaptive immune deficiencies (1, 164, 165), which results in an increased risk of opportunistic (helminths, *Citrobacter rodentium*) infections (166). Clinical studies in children have revealed a relationship between lower seroconversion rates associated with oral vaccines and kwashiorkor (11, 12), which has been associated with almost half of all deaths of children below 5 years of age (165). Moreover, previous findings have shown that PCD diet impairs adaptive immune response in humans and mice (82–85, 193). Using the Gn piglet model, our group has demonstrated that vaccinated kwashiorkor exacerbated human rotavirus-induced diarrhea and significantly increased fecal virus shedding titers, which coincided with suppression of multiple innate and adaptive immune responses (13, 23–25). We have also demonstrated that kwashiorkor Gn piglets vaccinated with an oral rotavirus vaccine had decreased protection rates against diarrhea and fecal virus shedding compared to their counterparts receiving a normal diet. These parameters were associated with impaired innate (including NK-cell function and levels of IFN-α, TNF-α, IL-12, and IFN-γ cytokines) and adaptive (T cells, IgA/IgG antibody titers and antibody-secreting cell numbers) immune responses (24, 25). These results demonstrated the effects of PCD diet on immune responses to human rotavirus infection and on vaccine effectiveness.

## Gnotobiotic Mouse vs. Gnotobiotic Pig Model of Kwashiorkor

**Figure 3:**
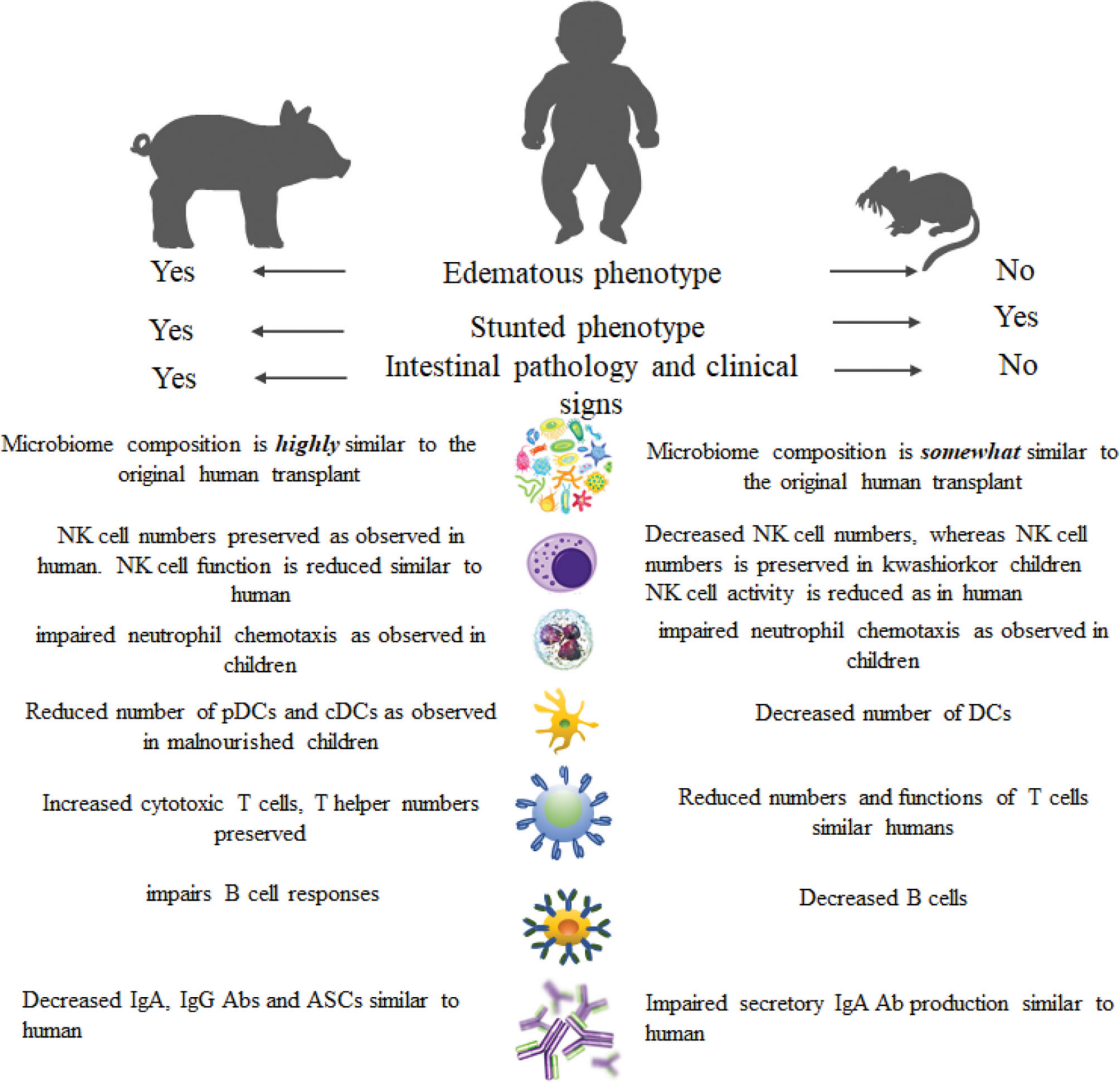
Comparison of clinical and immune parameters of kwashiorkor in humans, pigs, and mice.

Due to several intricacies and ethical concerns, it is not possible to conduct a human subject research to comprehensively study the impact of kwashiorkor on the host health, microbiome, and immune function. Thus, human microbiota-transplanted (microbiota humanized) animal models are used whereby selective microbial communities can be developed under controlled conditions. Moreover, animal models have provided novel insights into the immune function in malnutrition and highlighted various mechanisms (**Figure 3**). Several Gn animal models of kwashiorkor or protein/protein-calorie deficiency have been established (76, 194–196). It has been shown that decreased influenza Abs and cytotoxic T-cell responses were observed in mice fed a protein-deficient (PD, isocaloric) diet and infected with influenza virus compared with mice fed a protein-sufficient diet (76). These immune responses were reinstated following feeding the PD mice with a protein-sufficient diet (76). The PCD diet had led to undernutrition in mice leading to dysfunction of the gastrointestinal barrier characterized by reduced jejunal villus height which causes a reduction in nutrient absorption areas (194). The PCD diet reduced the protective efficacy by reducing antigen-specific IgA of the oral cholera and salmonella vaccines in a mouse model (195). Furthermore, there was an impairment of memory CD8^+^ T-cell proliferation in mice fed a PD diet following pathogen challenge (196). This study revealed the importance of the long-lasting and functional memory CD8^+^ T-cell populations for efficient recall responses to vaccine, and it suggests that long-term vaccine-specific immunity could be compromised in children with kwashiorkor. Moreover, Hickman and colleagues demonstrated that PD mice had increased weight loss, increased viral shedding, reduced levels of antiviral mucosal IgA, and prolonged clearance of norovirus compared to well-nourished mice (197). Surprisingly, kwashiorkor mice challenged with rotavirus had altered IgA responses to rotavirus vaccination and infection but not the impaired vaccine efficacy (198).

Mouse models do not fully recapitulate human physiology. Additionally, Gn mice transplanted with human microbiota did not support the growth of many major microbial taxa found in the original human donor stool (199, 200). While 99.3%–100% of genus-level taxa originating from human donor were found in the Gn pig passaged intestinal/fecal samples (14, 201, 202), only ˜85% of human microbiota genus-level taxa could be successfully transplanted into germ-free mice (199). Further, resulting microbial communities of Gn mice transplanted with human donor stool were enriched for phylotypes related to species of *Bacteroides*, whereas members of some *Clostridia* (*Firmicutes* phylum) clusters grew poorly in the mouse gut (203). Importantly, mouse models lack pathological changes and clinical signs seen in Gn piglets infected with human rotavirus, including the signature edematous phenotype of kwashiorkor (15, 204). Thus, Gn piglet transplanted with human intestinal microbiota is a clinically relevant model for studies evaluating the interactive impacts of nutrition, enteric pathogens, and the intestinal microbiota on gut function, immunity, and health of the host (205, 206).

Piglets are immunocompetent at birth, but immunologically immature (207). In pigs as in humans, secretory IgA Abs are prevalent in the milk, intestine, and mucosal secretions (208). However, swine placenta is impermeable to immunoglobulins, and thus neonatal piglets are born agammaglobulinemic ensuring no interference by maternal antibodies in surgically derived Gn piglets (209). Gn pigs infected with human rotavirus showed symptoms of transient viremia, diarrhea, and intestinal lesions similar to those observed in infants and young children (204). Further, because Gn pigs are sterile caesarian-derived animals and housed in isolators to assure their germ-free status, they allow for studies of gut colonization with single bacteria or transplantation with the complete fecal microbiota. Also, pigs are outbred animals, like humans and in contrast to various inbred mouse lines. Thus, Gn pigs represent a unique, clinically relevant model to investigate direct and indirect impacts of malnutrition on host metabolism, neonatal immune responses, enteric viral infections, or oral vaccine efficacy without interference from other confounding microbiota (208, 210). Moreover, as mentioned above transplantation of human infant fecal microbiota (HIFM) into Gn piglets recapitulates 99% of the infant microbial community which is unattainable in mouse models (13–16). Pigs are more closely related to humans than mice in terms of anatomy/physiology, genomics, immunology, protein sequence homology, omnivorous diet, and genetic heterogenicity (14, 15, 17). Thus, resulting humanized Gn pigs allow for control over multiple variables and the evaluation of gut responses that are not possible in human infants or mouse and other conventional animal models. Gn piglets have been comprehensively investigated to examine the impact of the intestinal microbiota on human rotavirus infection and vaccine efficacy (18–22). Moreover, we successfully established an HIFM-transplanted neonatal Gn pig model that recapitulates major aspects of kwashiorkor in children (**Table 2**) (13, 23). Using the kwashiorkor Gn pig model and human rotavirus infection, our group has shown that PCD diet leads to compromised innate and adaptive immune responses, impairing the gut epithelial barrier and leading to intestinal dysbiosis and altered amino acid homeostasis (13, 23–25). More recently, our group has shown reduced efficacy of human rotavirus vaccine using the Gn pig model of kwashiorkor (24, 25).

**Table 2:** Clinical parameters of kwashiorkor in children, piglets, and mouse.

Phenotypic characteristics	Children	Piglets	Mouse
Edema of neck, face, extremities, abdomen	✓	✓ (13)	–
Skin depigmentation	✓	✓ (13)	–
Sloughing	✓	✓ (13)	–
Hair thinning	✓	✓ (13)	✓ (49)
Inflammation	✓	✓ (13)	✓ (48)
Stunting	✓	✓ (13, 99)	✓ (48)
Weight loss	✓	✓ (13, 99)	✓ (29)
Growth failure	✓	✓ (13, 99)	✓ (49)
Loss of appetite	✓	✓ (99)	?
Anemia	✓	✓ (99)	✓ (28, 29)
Hepatomegaly	✓	?	?
Liver steatosis	✓	✓ (99)	✓ (48)
Hypoglycemia	✓	✓ (13)	?
Hypoproteinemia	✓	✓ (13)	?
Hypoalbuminemia	✓	✓ (13)	?
Hypotriglyceridemia/hypocholesterolemia	✓	?	?

?, unknown; -, not observed; ✓, observed.

## Conclusions

Substantial advancements have been made in our understanding of the kwashiorkor pathogenesis during intestinal infection and host defense mechanisms in recent years. PCD and the associated micronutrient deficiencies significantly increase the risk of infections due to impaired immunity. Immune cell activation and systemic proinflammatory mediator levels are increased in PCD which coincides with decreased immunoregulatory responses. Specifically, malnutrition impairs immune priming by DC in addition to decreased neutrophil, monocyte, and lymphocyte functions, which in turn leads to numerous perturbations in other innate and adaptive immune responses. Thus, therapies tailored to these complex immune impairments are needed to reverse/ameliorate the effects of childhood protein-calorie malnutrition.

A tri-directional relationship exists between diet, immune response, and microbiota. The functionality of many immune cells depends on host–microbiota metabolic pathways and interactions in addition to the protein and micronutrient availability. Undernourished children are more predisposed to infections, while chronic or repeated infections damage the digestive system impairing its ability to absorb nutrients, thus further aggravating malnutrition. Thus, the approaches to intervene kwashiorkor and the associated immune dysfunction require multi-pronged strategies that address the dietary, immunological, and gut function inadequacies. Clinical and immunological studies of children with kwashiorkor are difficult since the susceptibility and collection of clinical samples from these subjects are limited. Additional analyses of white blood cells and plasma could provide some information, but the examination of the host defense at the tissue/cellular level poses a major challenge for human subject studies. Moreover, clinical studies of immunological parameters of undernourished children in impoverished settings are challenging because of the reduced accessibility of research facilities and technologies. Innovative instruments for untargeted metabolomics, proteomics, microbiome–metabolome, and transcriptomics analyses are essential when applied to clinically relevant cohort studies. Finally, the discovery and validation of malnutrition biomarkers are essential for future clinical intervention studies in impoverished or limited-resource settings to identify subclinical malnutrition and evaluate the efficiency of various interventional approaches. To minimize the impact of the vicious cycle of malnutrition and infections, longitudinal studies are needed to identify and validate additional tools to mitigate certain infections, to evaluate the underlying immunological deficiencies, and to assess the success of the current nutritional interventions.

Mechanistic studies using animal models represent an excellent strategy and the only alternative to clinical studies; however, appropriate animal species for certain clinical and epidemiological aspects of malnutrition must be carefully selected. The appropriate animal models should be representative of the target age (infancy–childhood), developmental period, metabolism, microbiome, (mal)nutritional status, and susceptibility to the infection with target pathogens. Such models are essential because there is scarcity of knowledge of how milder (more prevalent form) malnourishment affects the immune function since majority of the studies are focused on acutely ill hospitalized malnourished children.

The Gn pig model highly resembles humans in terms of anatomy/physiology, genomic, protein sequence homology, immunology, omnivorous diet, and genetic heterogeneity and efficiently recapitulates human donor microbiome composition. Thus, Gn pigs represent a unique, clinically relevant model to investigate direct and indirect impacts of malnutrition on host metabolism, neonatal immune responses, enteric viral infections, or oral vaccine efficacy without confounding microbiota.

RUTF combined with antibiotics treatment is an internationally accepted standard therapeutic approach that was shown to increase body weight and reduce the mortality in malnourished children. However, the malnutrition-induced phenotypic deficiencies are only partially restored by the RUTF/antibiotic, thus suggesting lack of or incomplete gut microbiome restoration. Microbiome-directed complementary foods have been evaluated in preclinical animal studies that reestablished microbiome composition/function and increased biomarkers of normal growth and development as in healthy children. Thus, further studies that identify bacterial species and specific nutrients that can beneficially modulate the dysbiotic microbiome of malnourished children are of critical importance. Extensive research was done to explore the ability of various probiotics (mostly lactic acid bacteria) to improve the host health and immune response and to reduce gastrointestinal inflammation associated with different medical conditions and infectious diseases including HRV. “De Simone Formulation” is a probiotic formulation that improves intestinal epithelial function and improves symptoms in antibiotic-associated diarrhea and other conditions. Therefore, probiotics can be used to treat/prevent infections and beneficially modulate microbiome in malnourished children. The above therapeutics can be preclinically validated in the Gn pig model of kwashiorkor.

Finally, innovative immunological studies can educate how cell signaling pathways associated with certain nutrients can impact cell cycle and functions. These data can also shed light on how the effectiveness and the persistence of vaccine-specific immune responses can be specifically altered by malnourishment. Novel interventions and vaccine adjuvants derived from various beneficial microorganisms with immunomodulatory properties should be explored in the context of malnutrition and post-vaccination immune responses.

## Author Contributions

HM and AV conceptualized the idea. HM drafted the manuscript. JA, AV, GR, and LS edited and revised the manuscript. All the authors contributed to the article and approved the submitted version.

## Conflict of Interest

The authors declare that the research was conducted in the absence of any commercial or financial relationships that could be construed as a potential conflict of interest.

## Publisher’s Note

All claims expressed in this article are solely those of the authors and do not necessarily represent those of their affiliated organizations, or those of the publisher, the editors and the reviewers. Any product that may be evaluated in this article, or claim that may be made by its manufacturer, is not guaranteed or endorsed by the publisher.
